# “Am I gonna get in trouble for acknowledging my will to be safe?”: Identifying the experiences of young sexual minority men and substance use in the context of an opioid overdose crisis

**DOI:** 10.1186/s12954-020-00365-4

**Published:** 2020-03-30

**Authors:** Trevor Goodyear, Caroline Mniszak, Emily Jenkins, Danya Fast, Rod Knight

**Affiliations:** 1 British Columbia Centre on Substance Use, 400-1045 Howe St, Vancouver, BC V6Z 2A9 Canada; 2 School of Nursing, University of British Columbia, Vancouver, Canada; 3 School of Population and Public Health, University of British Columbia, Vancouver, Canada; 4 Department of Medicine, University of British Columbia, Vancouver, Canada

**Keywords:** Substance use, Overdose, Opioid, Harm reduction, Youth, Young men, Sexual minority men

## Abstract

**Background:**

North America and other parts of the globe are in the midst of a public health emergency related to opioid overdoses and a highly contaminated illicit drug supply. Unfortunately, there is a substantial gap in our understandings about how this crisis affects key populations not conventionally identified within overdose-related surveillance data. This gap is particularly pronounced for gay, bisexual, and other men who have sex with men (sexual minority men)—a population that experiences substance use-related inequities across adolescence and young adulthood.

**Methods:**

We draw on in-depth semi-structured interviews conducted in 2018 with a diverse sample (*N* = 50) of sexual minority men ages 15–30 who use substances and live in Vancouver, Canada, to identify how patterns and contexts of substance use are occurring in the context of the opioid overdose crisis.

**Results:**

Our analysis revealed three themes: awareness, perceptions, and experiences of risk; strategies to mitigate risk; and barriers to safer substance use. First, participants described how they are deeply impacted by the contaminated illicit drug supply, and how there is growing apprehension that fatal and non-fatal overdose risk is high and rising. Second, participants described how procuring substances from “trustworthy” drug suppliers and other harm reduction strategies (e.g., drug checking technologies, Naloxone kits, not using alone) could reduce overdose risk. Third, participants described how interpersonal, service-related, and socio-structural barriers (e.g., drug criminalization and the lack of a regulated drug supply) limit opportunities for safer substance use.

**Conclusions:**

Equity-oriented policies and programming that can facilitate opportunities for safer substance use among young sexual minority men are critically needed, including community- and peer-led initiatives, access to low-barrier harm reduction services within commonly frequented social spaces (e.g., Pride, night clubs, bathhouses), nonjudgmental and inclusive substance use-related health services, the decriminalization of drug use, and the provision of a safe drug supply.

## Introduction

North America and other parts of the globe are in the midst of a public health emergency related to opioid overdoses and a contaminated illicit drug supply. In Canada, approximately 4000 people died from an opioid overdose in 2017—a 33% increase in overdose deaths from 2016 [1]. While each region of Canada has been impacted by the overdose crisis, the province of British Columbia (BC) has experienced the highest number of deaths from illicit drug (comprised of controlled and illegal substances, including heroin, cocaine, MDMA, methamphetamine, and illicit fentanyl) overdoses with approximately 1489 suspected in 2018—representing a rate of 31 deaths per 100,000 individuals [2]. This crisis is directly linked to the highly toxic contaminated drug supply, in which fentanyl and other synthetic analogs (alone or in combination with other substances) were implicated in approximately 87% of illicit drug overdose deaths in BC in 2018—a stark increase from just 4% in 2012 [2, 3]. At a population level and as a consequence of the overdose crisis, life expectancy at birth has now declined in BC—after experiencing annual increases from 2000 to 2013—by 0.38 years from 2014 to 2016 [4]. As such, in 2016, the British Columbia Ministry of Health’s Provincial Health Officer declared a public health emergency in response to the overdose crisis.

Despite the availability of data identifying key socio-demographic (e.g., age, gender) attributes of those populations affected by a fatal overdose, there is a substantial gap in our understandings about how overdoses are occurring within other key populations not conventionally identified within the overdose surveillance data, including among young Two-Spirit, lesbian, gay, bisexual, transgender, and queer (2SLGBTQ+) people. Nevertheless, the broader substance use-related literature suggests that—in comparison to the general population of cisgender and heterosexual individuals—gay, bisexual, and other men who have sex with men (sexual minority men) experience disproportionately high rates of substance use (across almost every substance) throughout adolescence and young adulthood [5–12], as well as an increased likelihood of drug dependence and substance use disorders [7, 8, 13–16]. Sexual minority men also experience a heightened risk of both fatal and non-fatal drug overdose relative to heterosexual men [17, 18]. Furthermore, as BC’s illicit drug supply is highly contaminated with synthetic opioids (e.g., fentanyl), there is an increasing concern of overdose being reported by those using non-opioid-based substances (e.g., meth, cocaine) [19, 20]. For example, recent data from drug checking services in Metro Vancouver indicates that the proportion of fentanyl-contaminated illicit non-opioid substances has been rising, and between 2017 and 2018, fentanyl was detected in 5.9% of amphetamine or methamphetamine samples and in 2.1% of cocaine or crack samples submitted for testing [21]. As such, given the elevated rates of opioid and non-opioid substance use among young sexual minority men in the context of a highly contaminated illicit drug supply, there is increasing concern that overdose-related risks and outcomes will disproportionately affect young sexual minority men [18].

Despite the limitations with sexual minority men and overdose-related data, there is an emerging literature describing the contexts and patterns of substance use among young sexual minority men. For example, a variety of motivations for substance use among young sexual minority men have been described, including how the use of recreational substances (e.g., cocaine, MDMA, ketamine, methamphetamine, alcohol) can act as a means for helping people to feel comfortable, to mitigate social unease, and to socialize and gain confidence in meeting potential sexual and/or romantic partners [22–26]. A growing body of evidence now indicates that the elevated rates of the human immunodeficiency virus (HIV) and other sexually transmitted and blood-borne infections (STBBIs) among young sexual minority men are highly associated with the *sexualized use of substances*, i.e., intensive polysubstance use to maximize pleasure and sociability with sex partners—a practice colloquially known in Canada as “*Party ‘n Play*” (or “PnP”) and “*Chemsex”* in other regions (e.g., Europe) [10, 15, 22, 24, 27–30]. Within this context and in their sexual lives more broadly, the diverse and innovative strategies used by sexual minority men to mitigate sexual health harms (e.g., HIV, STBBIs) have been well documented [27, 31–33], though less is known about how substance use-related harms are mitigated, particularly in the context of a contaminated illicit drug supply.

Among people who use drugs more broadly, however, a growing body of literature has described the evolving harm reduction strategies used to mitigate overdose risk in the context of a highly contaminated drug supply. These strategies have included a breadth of behavioral changes, including not using substances alone (i.e., so that peers can assist in the event of an overdose), using one substance at a time, testing the potency of a substance by first using only a small amount, and using fentanyl test strips and other drug-checking technologies^1^ to screen for fentanyl contamination in illicit drug compounds [34–37]. Recent studies have also demonstrated the important role that trustworthy sources of substances play. For example, people who use drugs have turned to illicit prescription-based sources of substances and consistent drug dealers to procure substances of consistent quality and perceived safety [37–39]. Similarly, peer-based harm reduction initiatives have been identified as highly acceptable, effective, and trusted approaches to further engage people who use drugs in developing and taking up strategies to mitigate substance use-related harms, including overdose [40–42].

Despite the growing literature in this area, there remains a gap in our understandings about how the current opioid overdose crisis may be influencing the substance use contexts and behaviors of specific subpopulations of people who use drugs. Within the context of an increasingly contaminated drug supply, population-specific strategies that can reduce the harms associated with overdose are needed. The objective of this study is therefore to identify how patterns and contexts of substance use among young sexual minority men are occurring within the context of Metro Vancouver’s opioid overdose crisis.

## Methods

### Study overview

For the reader, it is helpful to consider our motivations and positionality with regard to our analysis. This research was conducted within a larger study examining how substance use and sexual behavior co-occur among young sexual minority men. The lead author of this research, a white cisgender (cis) gay man in his mid-20s, had previously witnessed through his personal experiences and through his practice as a youth mental health nurse how the substance use-related experiences and perspectives of young sexual minority men are shifting in the context of the opioid overdose crisis. Given the changing policy and healthcare landscapes associated with this public health emergency, our motivation for this research is to critically interpret how the patterns and contexts of substance use among this population can inform substance use-related policies and programs to more fulsomely reflect the health and social needs of young sexual minority men who use substances. As such, we frame this research within a modified grounded theory methodology and using a social constructivist interpretive framework that positions our past and current involvement with individuals, communities, and research as central to and inseparable from our analysis [43, 44]. To do so, we draw on critical theory and methods, including feminist practices of critical self-reflection to look beyond our own aforementioned interpersonal and clinical experiences and knowledge to inquire about how broader socio-structural conditions, environmental factors, and intersecting systems of oppression shape substance use- and overdose-related inequities [45], particularly for marginalized populations such as young sexual minority men.

### Study setting

This study was conducted in Metro Vancouver, BC, Canada, a metropolitan area with an approximate population of 2,463,431 people [46], among whom 304,630 are young men between the ages of 15 to 30 [47]. There are an estimated 27,183 men who have sex with men living within Metro Vancouver [48]. Metro Vancouver has been disproportionately impacted by the opioid overdose crisis and experiences the highest rate of illicit drug overdoses in the country [2]. The opioid overdose crisis also has distinct gendered impacts [49, 50], and in BC, men have accounted for 80% of all illicit drug overdose deaths between 2009 and 2019 [2]. Data regarding sexual minority populations and fatal and non-fatal overdose in Metro Vancouver are unavailable at this time.

### Sampling and recruitment procedures

Ethics approval for this study was obtained from the University of British Columbia Behavioural Research Ethics Board (#H16-01915-A013). Drawing on a stratified purposive sampling strategy [51, 52], we deliberately recruited specific subgroups of participants of varying social positions to obtain a heterogenous sample of young sexual minority men with diverse perspectives and experiences of substance use. Study participants were recruited from across Metro Vancouver through study advertisements posted in a variety of clinical and non-clinical sites that are easily and discretely accessible to youth (e.g., youth community health centers, youth sexual health clinics, washroom stalls at local coffee shops, community bulletin boards, bus stops), through community and research partner social media posts and on-site study advertisements, and through targeted advertising services on social media websites, such as Facebook and Craigslist. Upon becoming aware of the study and identifying their interest to the research team, prospective participants were eligible for inclusion if they were between 15 and 30 years of age; were fluent in English, lived within Metro Vancouver; self-identified as a man (including cis- and transgender- [trans] identified men) who has sex with men; were previously or currently sexually active (with any gender[s]); and had used substances with sex at some point in their lifetime. Participants provided written informed consent prior to data collection activities and were remunerated with a CDN$30 honorarium. Participants filled out an eighteen-item socio-demographic questionnaire.

### Data collection

Between January and December 2018, we conducted 50 in-depth, semi-structured interviews that lasted 1–2 h. Interview guides were designed to elicit how participants describe links between contemporary socio-cultural contexts, their sexual lives, and their understandings of substance use. For example, participants were asked to discuss their attitudes, practices, and decision-making strategies surrounding their use of substances in the context of Metro Vancouver’s opioid overdose crisis. Participants were also asked to share their insights on how the various health policies and programs they used, as well as other features of the healthcare system (e.g., accessibility of health-information and quality of interactions with service providers), influenced their health-related perceptions and practices.

### Data analysis

Interviews were audio-recorded, transcribed verbatim, anonymized and securely filed with identifying details removed, and managed using NVivo 12 qualitative software. A research assistant verified each transcript for accuracy. Initial analysis included reading and re-reading the accounts of young sexual minority men in our sample and organizing the data into patterns, which were then organized into substantive open codes that reflected the phenomena described by study participants [44]. Discrepancies between interpretations of initial codes and emerging themes were addressed through debriefing processes between study co-authors. In doing so, we were able to draw rich, contextualized findings about young sexual minority men’s substance use in the context of the opioid overdose crisis. After identifying central themes and corresponding sub-themes, our analytic strategy involved reflecting on and clarifying these themes in an iterative fashion to conceptualize themes that not only represent the *content* of the interviews, but also the *context* in which these stories were told. For example, we frequently returned to the interview transcripts, field notes, sociodemographic data, and audio files, as analyzing across these data sources provided additional detail and insights about various features of a social context that further clarified and reframed the themes that we present in this research.

## Results

In total, we interviewed 50 young sexual minority men for this study. Table 1 provides an overview of the socio-demographic and behavioral characteristics of the sample. Below, we offer our findings in three thematic sections: awareness, perceptions, and experiences of risk in the context of the opioid overdose crisis; strategies to mitigate risk; and barriers to safer substance use. We also present quotations from participant transcripts here to highlight key features of these narratives, and each quotation includes a short description of the participant’s socio-demographic profile, a simplified classification of their substance use profile (i.e., non-opioid substance use vs. both opioid and non-opioid substance use), a participant-created pseudonym, and a researcher-assigned numerical identifier.

**Table 1:** Characteristics of participants

Participants	50
**Age** (average, range)	**24.18** (18–30 years)
Ethnicity^1^	
*White*	**26** (52%)
*Aboriginal*^*2*^	**10** (20%)
*Latin American*	**7** (14%)
*South Asian*	**5** (10%)
*Southeast Asian*	**2** (4%)
*Chinese*	**2** (4%)
*Korean*	**2** (4%)
*Filipino*	**2** (4%)
*Arab/West Asian*	**2** (4%)
*Black*	**1** (2%)
*Japanese*	**1** (2%)
*Other*	**2** (4%)
Gender^3^	
*Cisgender man*	**44** (88%)
*Transgender man*	**4** (8%)
*Queer*	**4** (8%)
*Genderqueer*	**2** (4%)
*Non-binary*	**1** (2%)
Sexual orientation^3^	
*Gay*	**29** (58%)
*Bisexual*	**14** (28%)
*Pansexual*	**6** (12%)
*Queer*	**4** (8%)
*Straight*	**2** (4%)
*Asexual*	**1** (2%)
*Unsure*	**1** (2%)
*Other (incl. homoflexible)*	**2** (4%)
Opioid use	
*Non-opioid substance use* *Both opioid and non-opioid substance use*^*4*^	**40** (80%) **10** (20%)
Substances used in the previous 12 months	
*Alcohol*	**47** (94%)
*Cannabis*	**41** (82%)
*MDMA*	**25** (50%)
*Cocaine*	**20** (40%)
*Poppers*	**20** (40%)
*Mushrooms*	**20** (40%)
*Meth*	**18** (36%)
*Benzodiazepines* *Ketamine*	**11** (22%) **11** (22%)
*Erectile dysfunction drugs*	**10** (20%)
*GHB*	**9** (18%)
*Speed*	**9** (18%)
*T3s*^*5*^	**7** (14%)
*Crack*	**5** (10%)
*Heroin*	**5** (10%)
*Fentanyl*	**4** (8%)
*Opioids*^*6*^	**4** (8%)
*LSD*	**4** (8%)
*Methadone*	**3** (6%)
*Steroids*	**3** (6%)
*Ritalin*	**1** (2%)
*Others*^*7*^	**8** (16%)

^1^Some participants selected more than one ethnicity

^2^Although our sociodemographic survey included the term “Aboriginal,” which refers to the First Nations, Inuit, and Métis peoples, as defined by the Constitution Act of 1982, this term has traditionally been used in a generic sense and in legal contexts [53]. In this paper, we use the term “Indigenous,” which refers to first peoples internationally

^3^Some participants selected more than one gender identity and/or sexual orientation

^4^All participants who reported using opioids engaged in polysubstance use that included non-opioid substances

^5^T3s (Tylenol #3) contain acetaminophen, caffeine, and codeine

^6^Other physician-prescribed opioids than those listed here

^7^Other substances used by participants included: Prozac, Concerta, Adderall, Dexedrine, Methadrone, MxP, 2CE, and 2CC, 2C-T-7, 2C-B, 5-Me0-DMT, nn-DMT, 4-AcE-DMT, allylescaline, nitrous oxide (whippets)

### Awareness, perceptions, and experiences of the risk in the context of the opioid overdose crisis: “It’s like you’re playing Russian roulette”

As our interviews began, participants tended to describe a growing apprehension within their social and sexual networks that the overdose risk is high and rising. As participants elaborated, many emphasized that they felt the prevalence and severity of the contaminated illicit drug supply underscored a very real and present danger to everyone who uses illicit substances, not just those previously thought to be of high risk (e.g., people who inject drugs; people who use opioids). For example, one 29-year-old cis queer gay man who uses opioid and non-opioid substances described the impact of the opioid overdose crisis and contaminated drug supply:

Fentanyl scares the crap out of me. Like, it, like, literally scares… like, it literally shivers me to my bones that, like, some drug dealer could be cutting drugs with fentanyl and people are – could be dying. [. . .] It’s like you’re – you’re playing Russian roulette (W1-YM-041-Valentina).

As participants’ stories unfolded, some participants’ experiences with overdose risk were underscored by situations in which they had unintentionally consumed a substance contaminated with fentanyl. For example, one 26-year-old cis bisexual man who uses opioid and non-opioid substances described how he had engaged in sexualized use of cocaine that he later discovered was laced with fentanyl:

I remember having sexual activities with somebody and being really high. And I thought I only did cocaine . . . only to find out three days later when I – when I ended up in Detox that I was told that I did fentanyl. There was fentanyl in the cocaine (W1-YM-018 Victor).

Across our data, participants described how their heightened awareness of the opioid overdose crisis stemmed from their personal experiences with substance use and overdose. Some participants described how they had personally experienced an overdose, and, in some cases, how their peers and family members had passed away due to overdose. For instance, one 20-year-old trans gay man who uses non-opioid substances described how:

People are dying from, like, fentanyl, and, like . . . heroin, and, like, basically, any type of opiate really. [Small nervous chuckle] Um, I have dealt with that myself. Like, I lost my brother last year to it. [. . .] I’ve lost people to opiates, I’ve lost myself. Um, it’s just, like, it’s really bad. And, like, I wish people had the proper treatment to be able to wean themself [sic] off it, or . . . I don’t know. People just deserve better help than what they have right now (W1-YM-027 Samuel).

In summary, participants described being highly aware of and impacted by the contaminated drug supply. As such, these data begin to distil how substance use among the study sample—embedded within a context featuring a dangerous drug supply—is underpinned by a sense of a fear and urgency.

### Strategies to mitigate risk: “I’m definitely a lot more cautious”

#### Trustworthy sources

Almost all of the participants described how they employ a series of risk-reduction strategies to reduce the likelihood that they will unintentionally consume a contaminated substance. As participants elaborated on how they defined or operationalized a “trustworthy” source, several described how they preferred procuring their drugs from drug dealers who themselves used those drugs—and, particularly, when these drug dealers also were members of their peer and sexual networks. Participants described how these trusted drugs were assumed to have more genuine intentions and motivations, which they contrasted to the motivations of “untrustworthy” and unfamiliar drug dealers, such as those “off the street,” who were described as simply wanting to maximize their profits. For example, one 27-year-old cis bisexual man who uses opioid and non-opioid substances described how:

If I, like, bought off the street off someone random then I would want my drugs checked. But I have a really good source. [. . .] A lot of the people down here that sell drugs are, like, are users as well. So, I don’t know, I just trust the drugs from them a lot more (W1-YM-016-Anthony).

Several participants also emphasized how a “trustworthy” source corresponded to a long-term relationship that they had cultivated and now perceived as being “consistent.” At times, participants compared how various features of a relationship with a drug dealer could be analogous to that of a sexual relationship, including the implications around risks (e.g., STBBIs; contaminated drugs). For example, one 27-year-old cis bisexual man who uses opioid and non-opioid substances described features of a “trustworthy” dealer:

I usually go through one – I usually trust one person, I buy my drugs from him. A particular dealer. No multiple dealers. [. . .] You can easily get it [fentanyl] when you go from one dealer to the next. You know. When you stick to one dealer, it’s just – it’s just like sexual partners, to me, you know. When you stick to one sexual partner, there’s lesser risk of contracting any virus or disease. When you engage in multiple partners, there’s always a risk for transmitting diseases and virus. So, the same way it goes with drugs, too. I stick to one part-[ner] – one dealer, and it has – it has always worked – worked well for me (W1-YM-019-Ben Castro).

As participants elaborated on their evolving decisions and practices surrounding the procurement of illicit substances, many described increased concerns about the safety of the sources from which they acquire substances. As such, having a trustworthy source had become far more important because of the overdose crisis, and using drug dealers they did not have an established relationship with was no longer acceptable. For example, one 25-year-old cis gay man who uses opioid and non-opioid substances elaborated:

Maybe before [the overdose crisis] I would be more like lenient with who I bought from. Like, not from the same source. But now it’s like, I’ll buy from the same source because I know like, they probably like, at least know that their source doesn’t have it [fentanyl] in there, ‘cause I don’t think they would like, be selling it if it was like, you know, kind of . . . I don’t know, killing off their customers, so to speak! (W1-YM-028-Ken).

To reduce the risk associated with what many viewed as a potentially lethal “gamble” that they may unintentionally acquire potentially contaminated substances, participants also described using alternative sources from more conventional sources. Specifically, several participants described how their concerns of overdose risk had led them to using the darknet to procure their substances, and these participants tended to describe the darknet as a more “responsible” and “controlled” source. The darknet—an illicit online market that shares some characteristics with legitimate online marketplaces, where customers can search for and compare vendors and the quality and safety of substances [54]—was frequently described as having the potential for obtaining a drug supply that is both “clean” and “safe.” For example, one 18-year-old trans pansexual man who uses opioid and non-opioid substances described how, after he found out his previous dealer had laced his drugs with other substances, he began to procure substances from the darknet as a safer alternative:

The only place I’ll- I’ll get my drugs is from the darknet because . . . everything on the street is – isn’t – is- is laced with something, or it- it’s just cut and it gets stepped on and it’s not even good most of the time. And it’s over-priced – it’s way over-priced – everything on the street – everything that I’ve ordered off there [darknet] has been exactly as described and I’ve never had any problem with anyone on the darknet and everyone’s pretty nice on there, actually (W1-YM-026-Wall-e).

Those who had used the darknet for procuring substances viewed it is as a highly favorable alternative to more conventional in-person drug suppliers. These participants described the darknet sources as being highly sophisticated and that there were several “built-in” quality controls, including customer reviews. As such, participants described both the reasons they trusted the darknet, as well as the strategies they used on the darknet, to ensure they were procuring clean and safe drugs:

It’s almost like buying something on Amazon ‘cause the vendors on the – on the darknet – they have, like, trust ratings and every time someone orders something, they have to review them out of five stars and that’s how you find out if the vendor’s actually…good, because people will say, “Oh, I got my – I never got my shipment in the mail,” or, “I got my stuff but it was laced with something,” and, or people will say, “Oh, I got my stuff and it was really good.” [. . .] I feel like it’s more trustworthy than buying off the streets ‘cause you can actually see other people, what other people are saying and what they think, and if there’s a problem with whatever they’re selling then you don’t buy from them and you find someone else and . . . it's so much cheaper on the darknet and- [pause] it’s – and- and the stuff is actually better and clean and good because it’s coming from people that that’s, like, their life [chuckles]. That’s all they do (W1-YM-026-Wall-e).

A small subset of participants also described procuring substances from trusted sources in their peer and sexual networks. In these instances, participants described how peers and sexual partners in their networks would “take charge” and look after others by offering advice about safer drug use, anticipating and preparing for potential side effects, and providing supplies to prevent and mitigate adverse outcomes, such as overdose (e.g., by supplying fentanyl test strips, Naloxone kits, and bottles of water). For example, one 26-year-old cis gay man who uses non-opioid substances described how an older man in their sexual network held a position of authority in which he was perceived as an influential decision-maker and trusted source for clean meth and GHB:

There is kind of a large core group [within our sexual network] . . . I wouldn’t say he’s exactly the leader, but he’s the most [chuckles] . . . I guess, experienced one? In that sense of this person owns a house in [suburb of Metro Vancouver] and he . . . has a sex dungeon [laughs] in his basement. Uh, so it’s this very large space to play and he really takes care of everybody in terms of, like, having water, towels provided. Washrooms are all there, the douches are all there. Um, so . . . this person, he’s older than the rest of us. He . . . [sighs] I don’t know exactly – him making executive decisions is the right phrase to use. Um . . .maybe, like, he just has more influence on people and because he is . . . more established or more experienced in this sense, we trust him for his word. [. . .] So, it’ll usually be him and also, my, um . . . my past ex. Yeah. So, it’s usually the older . . . it’s usually, like, the older guys that tend to buy the drugs (W1-YM-029-Swell).

As such, within the context of a highly contaminated drug supply, these data illustrate the extent to which participants are enacting a variety of strategies for defining and operationalizing “trustworthy” sources of substances.

#### Enacting harm reduction strategies

Participants also described several harm reduction strategies they had “taken up.” Here, participants described how they have operationalized both individual and collective strategies to mitigate the harms associated with the opioid overdose crisis, including frequenting supervised consumption sites, using sterile injection supplies and practices, obtaining and using Naloxone kits, attending Naloxone training programs, reducing their frequency of use, altering the route of substance administration (e.g., smoking or ingesting a substance, rather than injecting it), testing the potency of a substance by initially using only a small amount, and using drugs with peers rather than alone. For example, one 27-year-old cis bisexual man who uses opioid and non-opioid substances, and one 29-year-old cis homoflexible man who uses non-opioid substances, described how they enact harm reduction strategies to reduce risk in individual and group settings:

When I use, like, heroin and stuff, I don’t shoot. Like, I don’t inject drugs. But, I mean, that, like, doesn’t really make it any [small chuckle] safer. Uh, but I’m definitely a lot more careful. Like, I smoke it myself so I’ll have, like, a little bit, and then I’ll . . . I’ll wait, like, between – I’ll do, like, two or three hits, and then give it five or 10 minutes, because some of it, you know, you’ll be doing it, you won’t feel it, and then five minutes later, all of a sudden it hits you like a shit brick house. And so yeah, yeah, I’m definitely a lot more cautious [since the opioid overdose crisis] (W1-YM-016-Anthony).

[The] idea is not using alone and not using at the same time. Um, uh, just ‘cause that way there’s someone else around you that if you were to go – for instance, with fentanyl, right? If you were to go into overdose, they could then administer a drug [Naloxone] to you. If you both used at the same [time], then there’s no one left to actually help either of you (W1-YM-040-Mark).

Several participants expressed interest in and described their previous experiences with drug checking services and fentanyl test strips as a means for promoting safer substance use. While many participants described already routinely screening their substances for contamination with fentanyl, other participants described how this was not standard practice, and how they would only do so if they were unsure of the quality and safety of their sources, such as if they had obtained substances from an unfamiliar supplier. For example, one 24-year-old cis bisexual man who uses non-opioid substances described the heightened overdose risk associated with the contaminated drug supply and then revealed how using fentanyl test strips can both alleviate his concerns and support him to use MDMA more safely:

I was at a point where I was . . . and I was telling everyone, you know, not to do it [MDMA] because it’s – the risks [of overdose] are too high with the fentanyl crisis that’s going on. [. . .] I am feeling a little bit more relaxed about it, but still not at a point where I would want to do that any kind of regularly at all. [. . .] I would wanna be really careful to know where it’s come from. And test it as well . . . to make sure it’s actually pure, and not, uh, cut with fentanyl, for example, right? (W1-YM-013-Archie Andrews).

As participants elaborated on the different strategies they use to reduce overdose risk, a small subset of participants described using an experimental subject strategy to promote safer substance use within their peer networks. In this approach, one member of a group would first consume the substance and the others would consume the substance after it was determined to be safe (i.e., if taking the substance did not cause an overdose to occur). For example, one 21-year-old cis gay man who uses non-opioid substances described how he applies this experimental subject strategy to manage risk during recreational drug use with peers:

I definitely feel like I am, like, [small chuckle] scared of it [overdose]. Like, the most recent time that I did MDMA, like, I only did it because, like, other people had done it, like, a couple hours before, and they weren’t, like, dead or anything [laughs] So, I feel like I am a little bit, like, scared about it, yeah. [. . .] Yeah, I think it makes me just, like, not do it unless, like, someone else has gotten them and they’ve already tried some and they . . . yeah, I think that’s it. I pretty much just, like, wouldn’t do it and wouldn’t try to acquire them – I pretty much would only do it if, like, my friend got some, and they felt confident about who they got it from, and they’ve, like, probably already had it (W1-YM-012-Hudson).

As these accounts demonstrate, participants are enacting a variety of harm reduction strategies, largely centered on leveraging their social connections, to mitigate the heightened overdose risk associated with the contaminated drug supply.

### Barriers to safer substance use: “Am I gonna get in trouble for acknowledging my will to be safe?”

Despite describing a variety of strategies to alleviate concerns about the potential for overdose, participants expressed continued apprehensiveness about the overdose crisis. For example, participants revealed how socio-structural, service-related, and interpersonal barriers constrain opportunities for safer substance use. Participants emphasized how the penal nature of substance use-related regulatory and legal frameworks unjustly restricts the opportunity to access uncontaminated substances. For example, one 24-year-old cis gay man who uses non-opioid substances described how:

[The darknet] literally had a menu with stock levels and info and dosages and . . . and it was kind of like “responsible drug dealing.” [. . .] If we were in a perfectly progressive society then that might be how it would operate, you know, if it was more taxed and more controlled and stuff (W1-YM-001-Joe Bloggs).

Participants further described how concerns over penalty limited opportunities and willingness to engage with drug-checking services. Within this context, participants described a reluctance to access healthcare facilities offering drug-checking services due to concerns that their substances could be confiscated by service providers or, worse, that disclosing they are in possession of illicit substances could result in drug-related criminal charges (e.g., possession of a controlled [illicit] substance). A 26-year-old cis bisexual man who uses non-opioid substances further elaborated on the tensions that exist between the law and harm reduction services—in turn, creating vulnerability for people who access these services:

To test the drugs, how do you know you’re not going to be put away [incarcerated] for having them? Um, [laughs] that’s a big thing, too, because you can go and get your drugs checked, but you don’t know what’s gonna happen after you get them checked. They’re either gonna get taken away or you’re gonna get mad because they got taken away. Or you’re gonna get arrested for acknowledging that you have the drugs. [. . .] Am I gonna get in trouble for acknowledging my will to be safe? You know, I . . . I think drug users have enough to worry about. They shouldn’t have to be worried about being put away because they want to be safe, you know? It’s kind of a double-handed . . . situation. [. . .] You’re not gonna condone the use but you’re gonna condone the will to want to use safely. It doesn’t make sense (W1-YM-033-Salvadore).

Several participants also described how barriers within health systems and services limit opportunities for safer substance use. Participant experiences revealed how harm reduction services are finite and supported by limited resources. Moreover, a subset of participants described how they were unaware of existing substance use-related services. For example, participants described that the current harm reduction intervention “landscape” was not viewed as being accessible or intended for them because of their sexual identities and the contexts in which they use substances (e.g., sexualized, recreational, episodic). For instance, one 29-year-old cis gay man who uses non-opioid substances described hesitancy to discuss their sexualized substance use with healthcare providers because of the potential for judgment related to their sexual identity and experiences:

Heterosexuals are uneducated sometimes, and [have] preconceived ideas about MSM [men who have sex with men] sort of sexuality or lifestyles. [. . .] I always am a little bit apprehensive at times, especially knowing if a nurse or doctor doesn’t have any sort of, like, training or sort of background working with LGBTQ sort of people. I always am a bit hesitant to share information about myself to get a positive or a good answer. I guess, yeah, just nervous about judgement or being put down about it (W1-YM-034 Bruce Smith).

Similarly, this participant further described how harm reduction services were perceived as being for people with *dependent*—rather than *recreational—*substance use (i.e., “not for them”):

I could definitely see myself using it [drug checking services] at somewhere more public, like, or at Pride or, like, that kind of thing. [. . .] Granted the environment is sort of, like, secluded or safe or that kind of thing. Um, for me Insite [supervised consumption site] always seems like a bit of a . . . it’s for, like, a very specific type of person and that resource is often really strained. [. . .] As someone who’s, like, a recreational drug user and not, like, an intravenous drug user, I don’t feel as comfortable going there because it’s just, like, you are spread so thin. [laughs] And, like, I’ve been in Insite asking for a Naloxone kit and they’re, like, “no.” [laughs]. . . Uh, ‘cause they were, like, “We’re sorry. Like, unless you’re an intravenous drug user, we can’t really give you that because we don’t have the resources to actually do it. Go here.” [. . .] I feel like Insite and a lot of, like, the sort of, like, drug user resources are very, you know, stretched thin and it’s, like, a little bit, um, less if you . . . if you want to, like, you know, like, bring that sort of service to, uh, more people. Like, it’s very stigmatized or, like, looked down upon by others (W1-YM-034 Bruce Smith).

As participants elaborated, they described additional challenges to safer substance use within recreational and sexualized contexts, including having limited time, opportunity, and competency (e.g., because of already being impaired by substances) to establish risk and enact harm reduction strategies with members of their peer and sexual networks. A small subset of participants described how peer pressure and social expectations made safer substance use particularly challenging when in social settings (e.g., night clubs, bathhouses, Pride events, and group sex parties) and during encounters with unfamiliar sexual partners—many of whom were encountered through social media (e.g., hook-up/dating apps). Within these settings and networks, participants described instances in which, although harm reduction supplies had been available, participants and their peers had limited knowledge about how to actually use them. For instance, one 26-year-old cis gay man who uses opioid and non-opioid substances recounted how:

Everybody there [at an after-hours club] does coke and it’s just all out in the open. It’s very casual. And they have, they recently introduced, like, Narcan [Naloxone] kits in there, so it’s, I mean, it’s a good thing. [. . .] Because it’s a party, people just buy drugs and people use them and . . . I don’t know if they’re, like, as worried. Um, yeah. I don’t know, but I guess because you’re in that atmosphere you just . . . are kind of in the mood to not care, really. And if something does happen, we know that . . . there’s a Narcan kit. But . . . does that mean that we know how to use it? Not exactly. We all assume that maybe just the coat check girl who’s hanging on to it probably knows how to use it. Or someone does hopefully (W1-YM-029-Swell).

Across our findings, participants depicted a context in which regulatory and legal frameworks, strained health systems and services, and the opioid overdose crisis and contaminated drug supply precipitate complex challenges and risks related to substance use and overdose. Participants further described how a series of socio-relational influences (e.g., social expectations, peer pressure, unfamiliarity with sexual partners) created additional barriers to safer substance use.

## Discussion

While data regarding the magnitude of overdose experienced by 2SLGBTQ+ people is sparse, a well-known interrelated series of individual (e.g., suicide, mental illness), social (e.g., violence, bullying, and rejection from family and friends), and structural (e.g., poverty, homophobia, transphobia, stigma) factors are known to put this population at disproportionately high risk of substance use-related harms [55–58]. Our study highlights the deeply personal and widespread impact of the opioid overdose crisis and contaminated drug supply on young sexual minority men in Metro Vancouver. Moreover, our findings reveal the growing fear within this population that overdose risk is high—notably among young sexual minority men who had not previously perceived themselves to be at risk (e.g., those who do not use opioids, who do not inject drugs, who use substances recreationally or episodically).

Within the context of the opioid overdose crisis, our findings highlight how young sexual minority men are resilient and willing and able to operationalize harm reduction practices (e.g., drug checking technologies, Naloxone kits, not using alone) to mitigate potential substance use-related harms, including overdose. In line with previous research in other substantive areas (e.g., syndemics of mental illness, discrimination, HIV, and other STBBIs) [59–65], our study identifies how sexual minority men engage in diverse protective processes (e.g., modifying individual and peer health practices, building social support networks) that promote resiliency and foster positive health outcomes in response to the health risks they face. Our study also revealed how similar protective and risk mitigation strategies are being taken up to reduce overdose risk, including a variety of behavioral interventions and accessing trusted drug supplies. These findings are consistent with other research that demonstrates how people who use drugs have a high degree of awareness and knowledge about overdose risk, as well as strategies to mitigate this risk [36, 37].

In the context of a highly contaminated illicit drug supply, many participants also described how they needed to engage in “safer” procurement practices. Some described using online drug markets or procuring through members of their peer and sexual networks who they perceived as safe and trustworthy. Consistent with previous research [39], participants also described purchasing from drug suppliers who themselves use and/or screen their substances for contamination with fentanyl before selling them (e.g., with fentanyl test strips). Similar to research with other populations who use substances [39, 66, 67], our findings reveal how the procurement of substances from in-person drug suppliers who were trusted, reliable, and with whom participants had cultivated long-term rapport was perceived as both a viable and highly regarded harm reduction strategy. Similarly, participants described how the procurement of substances from online drug markets (e.g., darknet) was likewise influenced by trust, which, in this context and as described elsewhere, was developed through product and supplier consistency, as well as information gathered through sophisticated quality control and review processes [68, 69]. Notably, however, these approaches to procuring substances do not safeguard against the contaminated drug supply, particularly given that even “trustworthy” sources may unknowingly provide substances containing fentanyl. Nevertheless, these trust-laden strategies reflect the nuanced, intimate, and relational nature of harm reduction approaches taken up by young sexual minority men—a population who has historically led and “taken up” innovations in harm reduction in the context of HIV prevention [27, 70] and who seemingly draw on those lessons in their approach to prevent overdose risks. Furthermore, our findings demonstrate how harm reduction norms and practices emerge socially through intimate relationships with informal social controls among peer and sexual networks (e.g., friends, trusted drug suppliers, “leader” sexual partners), underscoring the complex and grassroots nature of this population’s approach to harm reduction.

Within the context of the current study, the vast majority of participants are described using a series of personal and network-based strategies to prevent harms associated with the opioid overdose crisis. However, as described by others [31, 71], while modifying substance use-related practices can certainly reduce potential harms, including overdose, this approach to safer substance use requires social spaces, healthcare settings, and sociopolitical contexts that allow for these practices to be “taken up” and, at times, these spaces presented barriers for practicing harm reduction approaches. For instance, a subset of participants in our study described how either they perceived—or were explicitly told by healthcare staff—that harm reduction services were “not for them.” Indeed, our participants describe considerable challenges in accessing and utilizing available harm reduction services. These findings further demonstrate how the majority of substance use services and infrastructure do not adequately respond to the unique needs and diversity of 2SLGBTQ+ people [55, 72]. As we have argued previously [55], there is a critical need to increase accessibility and availability of substance use-related services that are nonjudgmental, inclusive, and welcoming for young sexual minority men and other 2SLGBTQ+ people. Furthermore, young sexual minority men will benefit from tailored services that are responsive to the unique socio-relational contexts in which they use substances (e.g., recreational, sexualized, in response to sexual minoritization, and marginalization) [15, 27]. For example, one promising strategy to more equitably deliver culturally appropriate substance use-related services to young sexual minority men could include the provision of additional funding and infrastructure for grassroots and peer-led programs [32, 73], particularly given that these community-led and contextually relevant strategies are already being productively “taken up” by members of this population.

Our findings identify how the lack of a safe and unadulterated drug supply has situated young sexual minority men such that they must attempt to mitigate overdose risk by procuring substances from sources they perceive as trustworthy (e.g., online drug markets, trusted drug dealers). In the absence of larger scale interventions, however, these strategies alone will not sufficiently curb the rate of overdoses. Thus, given the extent to which illicit fentanyl and synthetic analogs are found in the drug supply is strongly correlated with overdose rates [20, 74, 75], our findings illustrate the need for a safe and regulated drug supply. Moreover, our findings reveal how the criminalization of substances, people who sell them, and those who use them [39] has positioned young sexual minority men within a socio-legal context where they must balance competing priorities of safer substance use and avoiding persecution, including when accessing harm reduction services (e.g., drug checking). This barrier is especially concerning, as accurate knowledge of drug content is of critical importance in reducing overdose risk [39]. Given the detrimental effects of surveillance and policing on people who use substances [76], as well as the uniquely antagonistic histories of policing with 2SLGBTQ+ people [77, 78], we are expressly concerned that the current overdose crisis is manifesting in ways that may distinctly harm young sexual minority men who use substances. In solidarity with our colleagues, we call for political advocacy efforts and de facto decriminalization zones in areas heavily impacted by drug criminalization [39, 79], including in queer community spaces where young sexual minority men frequently use substances (e.g., night clubs, bathhouses, Pride events).

It is clear from our findings that the existing “status quo” set of interventions to prevent overdose risk is narrow in scope. While implementing and scaling up peer-led, outreach, and low-barrier harm reduction services (e.g., fentanyl test strips, drug checking technologies, Naloxone kit distribution) could facilitate broader access to young sexual minority men who may otherwise be hesitant to access these services due to concerns overcriminalization [39, 80, 81], the measures currently available and used by young sexual minority men in Vancouver do not address the root causes of overdose-related inequities. Consequently, we argue for urgent and significant structural intervention that includes the decriminalization of drug use and the introduction of an accessible, regulated, and safe drug supply.

This study provides rich insights from a heterogenous sample of young sexual minority men who are actively using substances and who enact diverse harm reduction strategies within the context of the opioid overdose crisis. Nevertheless, several limitations to the study design warrant a discussion. First, social desirability bias may have led to inaccurate, incomplete, or under-reported descriptions of substance use among participants, particularly given the sensitive and stigmatized nature of the topics under study. Second, although our stratified purposive sampling strategy led to a large and diverse group of participants enrolling in this study, our inclusion criteria required that all study participants have previously used substances with sex; therefore, these findings may not be representative for those who do not use substances with sex. Moreover, as all study participants were under the age of 30 and resided locally, our findings may not be as applicable to sexual minority men who are older and/or who live in other jurisdictions. Third, given the high rates of polysubstance use reported by participants in our sample, we were unable to identify potentially differential patterns and contexts of substance use between those who used different types of substances (e.g., opioid vs. non-opioid vs. both). Similarly, we did not fully explore how these experiences may vary across subpopulations of young sexual minority men who are distinctly impacted by marginalizing social and structural conditions (e.g., poverty, unstable housing, racism, colonialism). Future research should explore how young sexual minority men’s intersecting social identities (e.g., class, ethnicity, Indigeneity, ability) interact with various contexts and systems of oppression to influence patterns and contexts of substance use.

## Conclusion

Our findings reveal how young sexual minority men are responding to a highly contaminated drug supply, including by enacting a variety of individual and collective harm reduction practices. Despite expressing a growing sense of urgency to reduce overdose risk, and operationalizing a series of strategies to promote safer substance use, young sexual minority men face several key challenges and barriers, including peer pressure and social expectations, inadequate healthcare infrastructure to support 2SLGBTQ+ people who use substances, drug criminalization, and the lack of a safe drug supply. Taken as a whole, these findings underscore the extent to which the current state response to the opioid overdose crisis is failing young sexual minority men who use substances. Equity-oriented policies and programming that can facilitate opportunities for safer substance use among young sexual minority men are critically needed, including community- and peer-led initiatives, access to low-barrier harm reduction services within commonly frequented social spaces (e.g., pride events, night clubs, bathhouses), nonjudgmental and inclusive substance use-related health services, the decriminalization of drug use, and the provision of a safe drug supply.

## Data Availability

The data analyzed during the current study is not publicly available due to it containing information that could compromise research participant privacy and consent, but it is available from the corresponding author on reasonable request.

## Abbreviations

BC: British Columbia
cis: Cisgender
MSM: Men who have sex with men
STBBIs: Sexually transmitted and blood-borne infections
trans: Transgender
2SLGBTQ+: Two-Spirit, lesbian, gay, bisexual, transgender, and queer

## Footnotes

1 Drug checking technologies vary significantly according to testing methods (e.g., colorimetric reagents, chromatography, mass spectrometry), type of results produced (e.g., presence or absence of contaminants vs. quantitative information about the degree of contamination), and setting (e.g., home, clinical sites, mobile services) [34].
